# Extending the Minimum Information About BIobank Data Sharing Terminology to Describe Samples, Sample Donors, and Events

**DOI:** 10.1089/bio.2019.0129

**Published:** 2020-06-12

**Authors:** Niina Eklund, Ny Haingo Andrianarisoa, Esther van Enckevort, Gabriele Anton, Annelies Debucquoy, Heimo Müller, Linda Zaharenko, Cäcilia Engels, Lars Ebert, Michael Neumann, Joachim Geeraert, Veronique T'Joen, Hans Demski, Élodie Caboux, Rumyana Proynova, Barbara Parodi, Sebastian Mate, Erik van Iperen, Roxana Merino-Martinez, Philip R. Quinlan, Petr Holub, Kaisa Silander

**Affiliations:** ^1^ THL Biobank, Department of Public Health Solutions, Finnish Institute for Health and Welfare, Helsinki, Finland.; ^2^ IARC, Lyon, France.; ^3^ Department of Genetics, University Medical Center Groningen, University of Groningen, Groningen, The Netherlands.; ^4^ Helmholtz Zentrum München, Neuherberg, Germany.; ^5^ Belgian Cancer Registry, Brussels, Belgium.; ^6^ Diagnostic and Research Center for Molecular BioMedicine, Medical University of Graz, Graz, Austria.; ^7^ Latvian Biomedical Research and Study Center, Riga, Latvia.; ^8^ Charité, Berlin, Germany.; ^9^ DKFZ, Heidelberg, Germany.; ^10^ Interdisciplinary Bank of Biomaterials and Data Würzburg, University Hospital Würzburg, Würzburg, Germany.; ^11^ Faculty of Medicine and Health Sciences, University of Ghent/University Hospital Ghent, Ghent, Belgium.; ^12^ IRCCS Ospedale Policlinico San Martino, Genoa, Italy.; ^13^ Medical Centre for Information and Communication Technology, Universitätsklinikum Erlangen, Erlangen, Germany.; ^14^ Amsterdam UMC Biobank, Amsterdam University Medical Centers, Amsterdam, The Netherlands.; ^15^ Karolinska Institutet, Stockholm, Sweden.; ^16^ Digital Research Service, University of Nottingham, Nottingham, United Kingdom.; ^17^ BBMRI-ERIC, Graz, Austria.

**Keywords:** MIABIS, sample, sample donor, biobank, standardization, interoperability

## Abstract

***Introduction:*** The Minimum Information About BIobank data Sharing (MIABIS) was initiated in 2012. MIABIS aims to create a common biobank terminology to facilitate data sharing in biobanks and sample collections. The MIABIS Core terminology consists of three components describing biobanks, sample collections, and studies, in which information on samples and sample donors is provided at aggregated form. However, there is also a need to describe samples and sample donors at an individual level to allow more elaborate queries on available biobank samples and data. Therefore the MIABIS terminology has now been extended with components describing samples and sample donors at an individual level.

***Materials and Methods:*** The components were defined according to specific scope and use cases by a large group of experts, and through several cycles of reviews, according to the new MIABIS governance model of BBMRI-ERIC (Biobanking and Biomolecular Resources Research Infrastructure–European Research Infrastructure Consortium). The guiding principles applied in developing these components included the following terms: model should consider only samples of human origin, model should be applicable to all types of samples and all sample donors, and model should describe the current status of samples stored in a given biobank.

***Results:*** A minimal set of standard attributes for defining samples and sample donors is presented here. We added an “event” component to describe attributes that are not directly describing samples or sample donors but are tightly related to them. To better utilize the generic data model, we suggest a procedure by which interoperability can be promoted, using specific MIABIS profiles.

***Discussion:*** The MIABIS sample and donor component extensions and the new generic data model complement the existing MIABIS Core 2.0 components, and substantially increase the potential usability of this terminology for better describing biobank samples and sample donors. They also support the use of individual level data about samples and sample donors to obtain accurate and detailed biobank availability queries.

## Introduction

The concept of *Minimum Information About BIobank data Sharing* (MIABIS) was originally developed in 2012 by the Biobanking and BioMolecular Resources Research Infrastructure of Sweden to facilitate biobank and sample collection data sharing.^1^ MIABIS Core, further updated to version 2 in 2016, consists of components defining biobanks, collections, and studies on an aggregated level.^2^ The first version of MIABIS Core was ontologized in 2013 and is referred to as *Ontologized MIABIS* (OMIABIS).^3^ It was later combined with another biobanking ontology forming the current *Ontology for biobanking* (OBIB).^4^ The use of ontologies facilitates integration of data from multiple sources. The integrated data can then be used for retrieving data in queries in a structured and organized form.^3,4^ The ontologization of MIABIS Core version 2.0 is almost complete and the new ontologized attributes are available through OBIB. The MIABIS Core version 2.0 is currently used in different biobank registers and catalogs, that is, in the *Biobanking and Biomolecular Resources Research Infrastructure–European Research Infrastructure Consortium* (BBMRI-ERIC) *Directory*^5^ and the *U.K. Clinical Research Collaboration (UK CRC) Tissue Directory*,^6^ and is implemented as part of data models for biobank and research information management systems such as Molgenis.^7^ The development of MIABIS is currently coordinated by the *Common Service IT* (CS IT) operations of BBMRI-ERIC.^8^

Different organizations and research infrastructures^9–12^ have previously undertaken steps to allow data from multiple studies to be aggregated and made available for reuse and sharing for other purposes. In addition, several initiatives have focused on standardization of the collection of data and their definitions by use of ontologies and biomedical standards.^3,4,13–16^ Although these initiatives and organizations have made relevant progress in their area of expertise, the produced reference terminologies are often too specific for the intended designed needs or too elaborate and are thus not generic enough to cover cases outside their original focus.

The aim of this study was to extend the MIABIS standard by describing sample donors (research participant/patient/donor) and samples at an individual level, thus extending the aggregated view provided by the MIABIS Core components. The work is based on several use-cases from the field of biobanking, which have the following in common: the sharing of individual-level data about samples and sample donors to support interoperability between biobanks/catalogs, and to enable availability queries for suitable samples.

## Materials and Methods

### Defining the scope of work and collecting use-cases

The guiding principles used for developing the MIABIS *Sample* and *Sample Donor* components were laid out by a small team of experts from BBMRI-ERICs CS IT, and the work plan was approved by BBMRI-ERICs Management Committee.

Six distinctive guiding principles were established as the foundation for the *Sample* and *Sample Donor* component definition work:

1. The components are aimed only at samples of human origin.
2. The components are applicable to all types of samples and all sample donors (i.e., no sample-type-specific attributes), as extensions can later be introduced by domain-specific MIABIS modules.
3. Hierarchical structures between the different attributes in a component are not defined within MIABIS, except for basic attributes that link the components and specific structured attributes. Each use-case and each infrastructure can define the data structure as needed for their own purpose.
4. The sample component aims to capture information on the current status of samples stored in a given biobank. Sample handling and processing history are not included in this study.
5. Each component is independent and searchable on its own. There are no dependencies with other information sources, except between sample and sample donor.
6. Predefined data terminologies are used if they exist, including attributes already defined for the MIABIS Core 2.0, which could also be used for describing individual-level data.

After the guiding principles for the MIABIS extension were established, use-cases for developing the *Sample* and *Sample Donor* components were requested by BBMRI-ERIC from their representative National Nodes. In total, five use-cases were identified, on which we based the development work:

1. BBMRI-ERICs *Sample Locator*^8^ is a federated query tool that enables researchers to query biobanks' individual-level data stored in the *Connectors* of individual biobanks to find biobanks that host samples and/or sample-related data that comply with the researcher's needs. The MIABIS *Sample* and *Sample Donor* component will form the common data model for the *Connectors*, whereas the federated querying interface providing aggregate responses to the queries is beyond the scope of this article.
2. Vendor-Neutral Sample Exchange Format: The *UK CRC Tissue Directory*^6^ aims to help researchers to find suitable samples for their research related to different diseases. By defining a vendor-neutral data exchange format, the transfer of sample information is allowed between different software solutions, which can originate from open source and commercial vendors.
3. *RD-Connect*^17^ is a global infrastructure project that links up databases, registries, biobanks, and clinical bioinformatics data used in rare disease research into a central resource for researchers worldwide.
4. *Amsterdam UMC Biobank*^18^ is a university hospital biobank, located at Amsterdam Medical Centers, Amsterdam, the Netherlands, aiming to create a catalog based on sample-level data to make its samples findable and accessible (FAIR principles^19^).
5. *German Biobank Alliance*^20^ aims to establish the technical prerequisites and IT tools for a consistent search, application, and joint use of biosamples throughout Germany.

The use-cases were used in defining the scope and requirements of the work, and in selecting and defining the attributes, which best describe sample donors and samples at an individual level.

### Governance model and review process

The MIABIS *Sample* and *Sample Donor* development work followed a new governance model. In this model the MIABIS development work is distributed into dedicated working groups that specify the components for specific extensions and modules. Each module has an initial scope of work as part of its setup process. The scope of each module is defined through specified use-cases. Before a working group can initiate the development work, BBMRI-ERICs Management Committee approves the suggested scope and work plan of the module. Once the working group has produced the finalized suggestion, the extension module goes through an extensive iterative review process by external experts selected by BBMRI-ERIC until all the comments have been addressed thoroughly. The finalized suggestion is submitted to the Management Committee of BBMRI-ERIC for approval. For the work on the MIABIS extension described in this study, we followed this new MIABIS governance model.

### Development of the components

The extension work of MIABIS was coordinated by the Finnish Institute for Health and Welfare, and it followed similar working principles as described for the development of the MIABIS Core 2.0.^2^ The development work included thorough discussion through web meetings and e-mail, using a joint Wiki page for distributing the working materials. The draft suggestion for component attributes was based on a consensus achieved during the development work. We aimed to implement the modularity of different components defined already in MIABIS Core,^1,2^ and therefore existing attributes were used whenever possible.

The draft suggestion of MIABIS *Sample* and *Sample Donor* components was sent to various experts for review regarding the content and description of the attributes. Feedback was received from biobanking and IT experts from Belgium, Finland, Germany, Latvia, Malta, and the Netherlands. The updated proposal was submitted for further review by BBMRI-ERIC to several domain experts. Based on the expert panel's comments, obtained during January to February 2018, the proposal was revised following a new round of web meetings and e-mail discussions. The current proposal was approved by the BBMRI-ERIC Management Committee in April 2019.

## Results

The current extension describes a minimal set of standard attributes for defining samples and individuals (research participants, donors, or patients), further denoted as sample donors. We suggest registering time-linked attributes that are related to the sample or sample donor through separate *Event* components. These attributes can be, for example, different observations with time stamps related to them. The *Event* components can then be linked to the sample and/or to the sample donor, allowing for modeling context, that is, describing the circumstances that form the settings of events related to the sample/sample donor. Defining the concept of *Events* is a common approach used in clinical models such as *Fast Healthcare Interoperability Resources* (FHIR) created by *Health Level 7 International* (HL7)^21^ or *OpenEHR*,^22^ which is an open platform for industry specifications, models, and software for e-health.

### Sample donor, sample, and event components and their attributes

MIABIS 2.0 Core was extended with independent components describing individual-level information on *Sample Donor*, *Sample*, and a generic template for *Event* with a few case examples of use. The new MIABIS components were defined as follows:

1. Sample donor is a person who is a source of either a biological material or a digital representation of a biological entity such as an image. The existing OMIABIS definition for a sample donor was modified based on the Merriam-Webster Dictionary definition.^23,24^
2. A sample is a portion or quantity of biological material that is collected from a sample donor, or which is a digital representation of a biological entity of the sample donor, such as an image. The existing OMIABIS definition for a sample was extended based on the Merriam-Webster Dictionary definition.^23,25^
3. An event is something that happens in a given place and time and is related to the sample and/or sample donor. The definition was created based on the NCI Thesaurus.^26^

After the components were identified and defined, attributes to be included in the new components were described and organized to where they would fit best. The modular structure of MIABIS defined in the MIABIS 2.0 Core work^2^ was maintained. The suggested attributes for each component are presented in Tables 1–3.

**Table 1.** Attribute Definitions in the Sample Donor Component

Attribute code	Attribute name	Allowed values	Attribute description	Constraints	Cardinality
MIABIS-SAMPLEDONOR-01	Sample donor ID	Coded String	Sample donor ID. Unique ID code of the sample donor within the sample collection/biobank	Pseudonymized, alphanumeric	1
MIABIS-SAMPLEDONOR-02	Sex	List: male, female, unknown, undifferentiated	Biological sex of the sample donor		1
MIABIS-SAMPLEDONOR-03	Data categories^a^	List: biological samples, survey data, imaging data, sample donor ethnicity, medical records, national registries, genealogical records, pathology records, physiological/biochemical measurements, psychological data, other	The data categories from which data are available or can be linked to the sample donor. Can be several values (See Supplementary Table S1 “Example on how to tabulate list-attributes for database use” for coded items).		0 … *n*
MIABIS-SAMPLEDONOR-04	Birth date	yyyy-mm-ddThh:mm:ss	Birth date of the sample donor. Coding ISO8601. Can also be partial, for example, YYYY.	Date of birth is required when Event date (MIABIS-EVENT-02) is used, otherwise partial date, as in birth year, can be used	0

^a^ See additional discussion in Synchronization with MIABIS Core section.

ISO, International Organization for Standardization; MIABIS, Minimum Information About BIobank data Sharing.

**Table 2.** Attribute Definitions in the Sample Component

Attribute code	Attribute name	Allowed values	Attribute description	Constraints	Cardinality
MIABIS-SAMPLE-01	Sample ID	Coded String	Unique ID of the sample within a sample collection, often represented by the sample barcode; text identifier. Sample ID meant for sharing	Pseudonymized, alphanumeric. It is recommended that sample IDs will be persistent within a given biobank	1
MIABIS-SAMPLE-02	Detailed sample type	Amniotic fluid; Ascites fluid; Bile; Body cavity fluid; Bone; Bone marrow aspirate; Bone marrow plasma; Bone marrow, whole; Breast milk; Bronchoalveolar lavage; Buffy coat; Cancer cell lines; Cerebrospinal fluid; Cord blood; Dental pulp; Digital sample; DNA; Embryo; Entire body organ; Feces; Fetal tissue; Fibroblasts; Gas, exhaled ( = breath); Gastric fluid; Hair; Immortalized cell lines; Isolated microbes; Menstrual blood; Nail; Nasal washing; Pericardial fluid; PBMC; Placenta; Plasma; Pleural fluid; Primary cells; Postmortem tissue; Proteins; Red blood cells; RNA; Saliva; Semen; Serum; Sputum; Stem cells and iPS cells; Swab; Sweat; Synovial fluid; Tears; Teeth; Tissue (Frozen); Tissue (FFPE); Umbilical cord; Urine; Urine sediment; Vitreous fluid; Whole blood; Whole blood, dried (e.g., Guthrie cards)	The sample type saved from a biological entity for testing, diagnostic, propagation, treatment or research purposes		1
MIABIS-SAMPLE-03	Sample storage temperature^a^	List: RT, 2°C to 10°C, −18°C to −35°C, −60°C to −85°C, < −135°C, Liquid nitrogen vapor phase, Liquid nitrogen liquid phase, Other	The long-term storage temperature at which the sample is stored after preparation, based on SPREC v3.		0
MIABIS-SAMPLE-04	Sample creation date and time	yyyy-mm-ddThh:mm:ss	The date and time the sample was created in the form currently described in MIABIS-SAMPLE-02 Detailed material type. Format according to ISO8601. Could also be partial, for example, YYYY.		0
MIABIS-SAMPLE-05	Anatomical site ontology	String	Name of ontology used for describing the anatomical source of the sample material, for example, ICD-O-3 topography code	MIABIS-SAMPLE-05 and MIABIS-SAMPLE-06 are required if any ontology information is provided	0/1
MIABIS-SAMPLE-06	Anatomical site ontology version	Coded String	Version of selected ontology for anatomical site.	MIABIS-SAMPLE-05 and MIABIS-SAMPLE-06 are required if any ontology information is provided	0/1
MIABIS-SAMPLE-07	Anatomical site ontology code	Coded String	Anatomical site code from the selected anatomical site ontology version	MIABIS-SAMPLE-05 and MIABIS-SAMPLE-06 are required if any ontology information is provided	0
MIABIS-SAMPLE-08	Anatomical site ontology description	String	Description from the selected anatomical site ontology code	MIABIS-SAMPLE-05 and MIABIS-SAMPLE-06 are required if any ontology information is provided	0
MIABIS-SAMPLE-09	Anatomical site free text	String	Explanation about Anatomical site in case of unknown Anatomical site or insufficient information	MIABIS-SAMPLE-05 and MIABIS-SAMPLE-06 are required if any ontology information is provided	0
MIABIS-SAMPLE-10	Sample content diagnosis	List: Healthy, [ICD-10 code], Unknown, Not applicable	The ICD-10 diagnosis code describing content of the sample, such as whether the sample contains cancerous material		0 … *n*
MIABIS-SAMPLE-11	Use restrictions	List: Commercial use restriction, DNA use restriction, Outside EU access restriction, Xenograft restriction, Other animal work restriction, Other restriction	The restrictions that may change the availability of the samples donated by the sample donor (see Supplementary Table S1. “Example on how to tabulate list-attributes for database use” for coded list items).		0 … *n*

^a^ See additional discussion in Synchronization with MIABIS Core section.

EU, European Union; FFPE, formalin-fixed, paraffin embedded; PBMC, peripheral blood mononuclear cells; RT, room temperature; SPREC, Sample PREanalytical Code.

**Table 3.** Generic Structure for Creating Event Records

Attribute code	Attribute name	Allowed values	Attribute description	Constraints	Cardinality
MIABIS-EVENT-01	Event ID	Coded String	Random ID for each event, created by the database implementation	Required if event is recorded	1
MIABIS-EVENT-02	Event date and time	yyyy-mm-ddThh:mm:ss	The date and time of the event. Coding ISO8601. Can also be partial, for example YYYY	Use either age at event or event date and time, not both. Date of birth is required when event date is used	0
MIABIS-EVENT-03	Age at event	Decimal	Age at the time of the event	Use either age at event or event date and time, not both	0
MIABIS-EVENT-04	Age at event unit	List: years, months, weeks, days, gestational weeks	Unit defining age at event	When age at event is provided, age unit is required	0

Tables 1–3 describe the detailed attribute and data item lists for *Sample Donor*, *Sample*, and *Event* components. The *Sample* component (Table 2) attributes *Detailed Sample Type* and *Sample Storage Temperature* are based on *the Sample PREanalytical Code* (SPREC), version 3^27,28^ standard with certain modifications and additions. The SPREC (version 3) attribute *Type of sample* was used to establish the initial list of values for the new MIABIS attribute *Detailed sample type*. Because SPREC includes only information on primary samples, we have added several values for processed samples that are commonly stored in biobanks, such as DNA and RNA, as well as the values *Digital sample* and *Postmortem sample*. We also edited the SPREC value list to omit information on sample processing method, which is not part of MIABIS. For example, the SPREC values *Plasma, single spun* and *Plasma, double spun* have been merged into a single value *Plasma* in MIABIS. Finally, we added a new structured attribute, *Anatomical site*, to provide information about the anatomical source of the sample material.

We also suggest an attribute called *Sample content diagnosis*, which reflects the content of the sample, that is, whether the sample contains cancerous tissue or not or whether the sample is taken from a tissue that is affected by a given disease. The *Sample content diagnosis* should contain the specific disease code/s if the sample is taken from a tumor and contains cancerous cells, or from a tissue that is affected by a disease.

All the components presented in Tables 1–3 can be readily used. However, some attributes allowing multiple values to be recorded for one item might require additional processing when implemented in database solutions. Such an example is presented in Supplementary Table S1, where each allowed value for MIABIS *Sample* component attribute *Use restrictions* is tabulated as an independent attribute receiving values Yes/No/Not applicable. In addition, the *Event* component is a generic template comprising only of the minimal items that should always be registered for an event (Table 3), and it should be complemented by carefully selected event-specific attributes. In this study, the *Event* component is kept short on purpose, because of plethora of different event types. To keep the MIABIS terminology and data model as general as possible, we only created a generic template for *Events* that can be used to define specific *events* for different purposes. To demonstrate the use of the *Event* component, we describe three use-case examples for events: *Sampling Event*, *Disease Diagnosis Event*, and *Death Event*, which are presented in Supplementary Tables S2, S3, S4.

### Changes in MIABIS structure to enhance interoperability

**FIG. 1.**
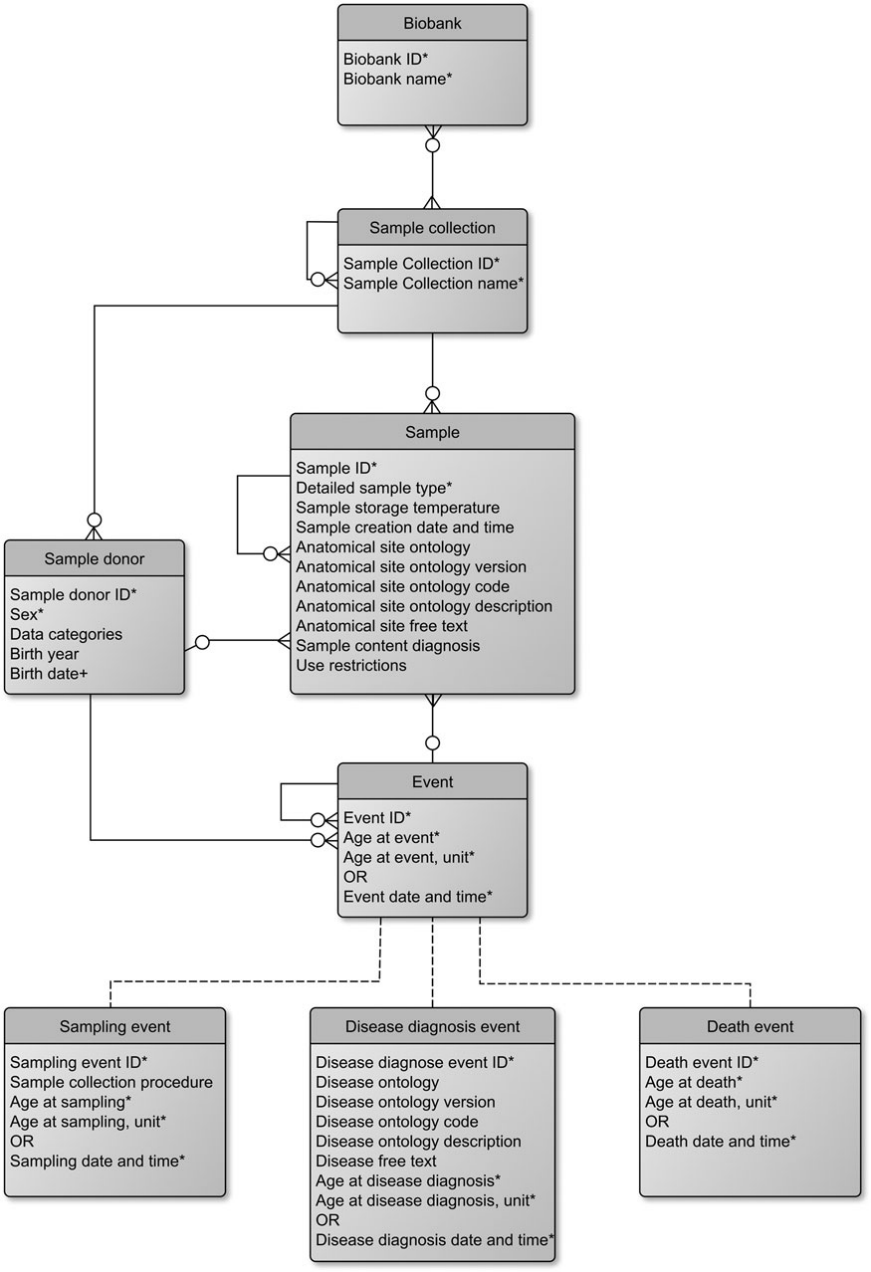
The generic data model for the MIABIS “Sample,” “Sample Donor,” and “Event” components, and how it is linked with the MIABIS Core components “Sample Collection” and “Biobank.” This diagram uses the Crow's foot notation. Different types of events can be linked to the model. Examples of these events are connected to the generic event with *dashed lines*, and they can be used to replace the “Event” component, shown as a placeholder. The required attributes in each component are marked with an *asterisk*. In addition, attribute “Birth date” in “Sample donor” component is marked with a plus to better distinguish its use-case dependency and the requirement to be implemented only when, that is, exact “Event date and time” attribute is used. MIABIS, Minimum Information About BIobank data Sharing.

Along with the MIABIS extension, a generic data model to support interoperability between biobanks sharing their individual-level data on samples and sample donors is suggested (Fig. 1). The data model was held very generic to ensure its usability for a variety of use-cases. To facilitate interoperability between information systems using MIABIS, and to add measures to improve their technical implementations, we suggest using use-case-specific profiles. These profiles may apply different restrictions in local implementations of the MIABIS data model. The profiles can enforce additional restrictions on relations to entities and turn some entities mandatory. For example, a profile can define 1…*n* cardinality, where the generic model only defines 0…*n*, such as requiring that for each sample minimal information on sample donor must also be provided, or a profile can include fixed or restricted values for an attributes, such as requiring that the sample donor's age is given in years.

To ensure that all use-cases are able to implement the MIABIS data model and can map their data to it without imposing any need for additional adjustments, we have used unique identifiers for each attribute and a component-specific prefix. Furthermore, for each attribute only one value is allowed. An example on tabulating list items as independent attributes is presented in Supplementary Table S1.

### Synchronization with MIABIS Core

The development of MIABIS *Sample* and *Sample Donor* components resulted in several small changes in MIABIS Core attributes, such as in *Data categories*, where new types of data were inserted, and *Sample storage temperature*, where the value *Liquid nitrogen vapor phase* was introduced to better distinguish the different temperature ranges associated with liquid nitrogen. Other changes include the coding scheme of attributes and adjustments in the data model. Furthermore, with the latest component additions, MIABIS is heavily focused around biological samples. However, some biobanks may not have biological samples, but are rather built around data. Such data-driven biobanks are becoming more common while biobank samples are converted into data, and new digital sample types emerge through biological imaging techniques. Thus, the concepts *digital samples* and *data-driven biobanks* need to be accommodated. Owing to these reasons, MIABIS Core is currently being updated into its third version, and work on this was initiated in May 2019.

## Discussion

The MIABIS *Sample donor*, *Sample*, and *Event* components were defined according to a specific scope and use-cases by a large group of experts, and through several cycles of review according to the new MIABIS governance model. The component extensions and the new generic data model complement the existing MIABIS 2.0 Core components, and substantially increase the potential usability of this terminology for describing biobanks and enabling searches based on individual samples. We believe that the current MIABIS extension is consistent with the use-cases defining the framework of the described development work. Additional modifications to the current MIABIS components will be carried out based on feedback from different biobank availability services, such as Sample Locator^8^ and UK CRC Tissue Directory,^6^ which are currently working on implementing the MIABIS profiles for their specific use-case. The proposed model has been used for a pilot version of the Locator service currently in operation by the BBMRI-ERIC German National Node, to validate the model in practical settings.

We identified attributes that were not generic enough or were beyond the scope of this study. These attributes, which are about sample quality and/or pre-analytical information, sample donor-related clinical data, and disease-specific sample data, are currently listed as possible new MIABIS extensions. However, for sample-specific pre-analytical information there are existing standards available, which we suggest to follow: SPREC,^27,28^ standards prepared by the *European Committee for Standardization* (CEN) working group on Technical Specifications for Pre-examination Processes (CEN/TC 140),^29^ or the *International Organization for Standardization* (ISO) standard on Medical laboratories—Requirements for quality and competence (ISO15189).^30^

We plan to follow the trend set by previous MIABIS work and to ontologize the MIABIS *Sample*, *Sample donor*, and *Event* components into OBIB. In addition, to increase interoperability, we aim to map the MIABIS data model to widely used electronic health resource standards, such as FHIR,^21^ OpenEHR,^22^ or the *Observational Medical Outcomes Partnership* common data model.^31^ Mapping MIABIS to other data standards is carried out to enable the direct use of distinct database tools together with MIABIS. Some biobanks may have databases that are established on already existing data standards, which is the case especially for biobanks in hospital settings. By mapping MIABIS terms to the existing clinical standards, the biobanks can effortlessly produce data in MIABIS format without having to impose large changes in their own database systems. Example for mapping between FHIR and MIABIS is MIABIS-SAMPLE-07, *Anatomical site ontology code*, which can be mapped to FHIR term *collection.bodySite.coding.code*. Further mapping of MIABIS terminology to these clinical standards is carried out in close collaboration with the BBMRI-ERIC National Nodes and their specific requirements.

## Supplementary Material

Supplemental data

Supplemental data

Supplemental data

Supplemental data
