# Supplementary material for: Extending the Minimum Information About BIobank Data Sharing Terminology to Describe Samples, Sample Donors, and Events
Source: Biopreserv Biobank. 2020 Jun 12;18(3):155–64. doi: 10.1089/bio.2019.0129 (PMC7310316; doi:10.1089/bio.2019.0129)
Supplement: Supplemental data [file Suppl_TableS1.pdf]

Supplementary Data

SUPPLEMENTARY TABLE S1. EXAMPLE ON HOW TO TABULATE LIST-ATTRIBUTES FOR DATABASE USE

Item code	*Item name*	*Allowed values*	*Attribute description*
MIABIS-SAMPLE-11-01	Commercial use restriction	List: yes, no, not applicable	Whether the use of the samples for commercial use is restricted
MIABIS-SAMPLE-11-02	DNA use restriction	List: yes, no, not applicable	Whether use of sample for DNA extraction and analysis is restricted
MIABIS-SAMPLE-11-03	Outside EU access restriction	List: yes, no, not applicable	Whether the sample can be used for research outside EU
MIABIS-SAMPLE-11-04	Xenograft restriction	List: yes, no, not applicable	Whether there is restriction on implantation of human cells within an animal to grow
MIABIS-SAMPLE-11-05	Other animal work restriction	List: yes, no, not applicable	Whether there is restriction on mixing the human cells with animals/animal cells
MIABIS-SAMPLE-11-06	Other use restrictions	List: yes, no	Other use restriction applies for the samples
