# Supplementary material for: Extending the Minimum Information About BIobank Data Sharing Terminology to Describe Samples, Sample Donors, and Events
Source: Biopreserv Biobank. 2020 Jun 12;18(3):155–64. doi: 10.1089/bio.2019.0129 (PMC7310316; doi:10.1089/bio.2019.0129)
Supplement: Supplemental data [file Suppl_TableS2.pdf]

Supplementary Table S2. Sampling Event as an Example on Events

Attribute code	*Attribute name*	*Allowed values*	*Attribute description*	*Constraints*	*Cardinality*
MIABIS-SAMPLING-01	Sampling Event ID	Coded string	Random ID for each event, created by the database implementation		1
MIABIS-SAMPLING-02	Sampling date and time	yyyy-mm-ddThh:mm:ss	Sampling date and time. The date and time when the primary (original) sample is taken. Format according to ISO 8601. Could also be partial, for example YYYY	Use either age at sampling or sampling date and time, not both	0
MIABIS-SAMPLING-03	Age at sampling	Decimal	Age of person in years at the time the sample was donated	Use either age at sampling or sampling date and time, not both	0
MIABIS-SAMPLING-04	Age at sampling unit	List: years, months, weeks, days, gestational weeks	Unit defining age at sampling	When age at sampling is provided, age unit is required	0
MIABIS-SAMPLING-05	Sample collection procedure	List: surgical procedure, biopsy (procedure), phlebotomy (procedure), other	The procedure that was used to extract the primary sample		0
