# Supplementary material for: Extending the Minimum Information About BIobank Data Sharing Terminology to Describe Samples, Sample Donors, and Events
Source: Biopreserv Biobank. 2020 Jun 12;18(3):155–64. doi: 10.1089/bio.2019.0129 (PMC7310316; doi:10.1089/bio.2019.0129)
Supplement: Supplemental data [file Suppl_TableS3.pdf]

Supplementary Table S3. Disease Diagnosis Event as an Example on Events

Attribute code	*Attribute name*	*Allowed values*	*Attribute description*	*Constraints*	*Cardinality*
MIABIS-DIAGNOSIS-01	Disease Diagnosis Event ID	Coded String	Random ID for each event, created by the database implementation		1
MIABIS-DIAGNOSIS-02	Disease diagnosis date and time	yyyy-mm-ddThh:mm:ss	Sampling date and time. The date and time when the primary (original) sample is taken. Format according to ISO 8601. Could also be partial, for example YYYY	Use either age at diagnosis or diagnosis date and time, not both	0
MIABIS-DIAGNOSIS-03	Age at disease diagnosis	Decimal	Age of person in years at the time of disease diagnosis	Use either age at diagnosis or diagnosis date and time, not both	0
MIABIS-DIAGNOSIS-04	Age at disease diagnosis unit	List: years, months, weeks, days, gestational weeks	Unit defining age at disease diagnosis	When age at disease diagnosis is provided, age unit is required	0
MIABIS-DIAGNOSIS-05	Disease ontology	String	Name of ontology used for disease, for example, ICD, SNOMED	MIABIS-DIAGNOSIS-05 and MIABIS-DIAGNOSIS-06 are required if any ontology information is provided	0/1
MIABIS-DIAGNOSIS-06	Disease ontology version	Coded String	Version of selected ontology for disease, for example, ICD-9, ICD-10, SNOMED-CT	MIABIS-DIAGNOSIS-05 and MIABIS-DIAGNOSIS-06 are required if any ontology information is provided	0/1
MIABIS-DIAGNOSIS-07	Disease ontology code	Coded String	Disease code from the selected disease ontology version, for example, C61	MIABIS-DIAGNOSIS-05 and MIABIS-DIAGNOSIS-06 are required if any ontology information is provided	0
MIABIS-DIAGNOSIS-08	Disease ontology description	String	Description from the selected disease ontology code, for example, malignant neoplasm of prostate	MIABIS-DIAGNOSIS-05 and MIABIS-DIAGNOSIS-06 are required if any ontology information is provided	0
MIABIS-DIAGNOSIS-09	Disease free text	String	Explanation about disease or symptom in case of unknown disease or insufficient information		0
