# Supplementary material for: Extending the Minimum Information About BIobank Data Sharing Terminology to Describe Samples, Sample Donors, and Events
Source: Biopreserv Biobank. 2020 Jun 12;18(3):155–64. doi: 10.1089/bio.2019.0129 (PMC7310316; doi:10.1089/bio.2019.0129)
Supplement: Supplemental data [file Suppl_TableS4.pdf]

Supplementary Table S4. Death Event as an Example on Events

Attribute code	*Attribute name*	*Allowed values*	*Attribute description*	*Constraints*	*Cardinality*
MIABIS-DEATH-01	DeathEventID	Coded String	Random ID for each event, created by the database implementation		1
MIABIS-DEATH-02	Death date and time	yyyy-mm-ddThh:mm:ss	Date and time of death incidence. Format according to ISO 8601. Could also be partial, for example YYYY	Stored only by biobanks, data not meant for sharing	0
MIABIS-DEATH-03	Age at death	Decimal	Age of person in years at the time of death	When age at death is provided, age unit is required	0
MIABIS-DEATH-04	Age at death unit	List: years, months, weeks, days, gestational weeks	Unit defining age at death	Required if age at death is provided	0
